# 4-Methyl-1-[4-(methyl­sulfan­yl)benzyl­idene]thio­semicarbazide

**DOI:** 10.1107/S1600536811028029

**Published:** 2011-07-16

**Authors:** Xiao-Gang Mu, Xuan-Jun Wang, Jing-Jing Yang

**Affiliations:** a No. 503 Faculty, Xi’an Research Institute of High Technology, Hongqing Town., Xi’an 710025, People’s Republic of China

## Abstract

The title compound, C_10_H_13_N_3_S_2_, is roughly planar (r.m.s. deviation = 0.086 Å). In the crystal, N—H⋯S hydrogen bonds link the mol­ecules into (001) sheets.

## Related literature

For a related structure, see: Li & Jian (2010 ▶).
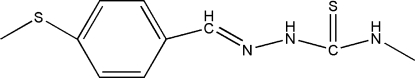


## Experimental

### 

#### Crystal data

C_10_H_13_N_3_S_2_
*M* *_r_* = 239.35
Monoclinic, 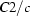

*a* = 14.123 (3) Å
*b* = 7.7789 (16) Å
*c* = 21.384 (4) Å
β = 96.31 (3)°
*V* = 2335.1 (8) Å^3^
*Z* = 8
Mo *K*α radiation
μ = 0.43 mm^−1^
*T* = 293 K
0.23 × 0.18 × 0.17 mm

#### Data collection

Bruker SMART CCD diffractometer
10756 measured reflections
2680 independent reflections
2368 reflections with *I* > 2σ(*I*)
*R*_int_ = 0.043

#### Refinement

*R*[*F* ^2^ > 2σ(*F* ^2^)] = 0.057
*wR*(*F* ^2^) = 0.170
*S* = 1.35
2680 reflections
136 parameters
H-atom parameters constrained
Δρ_max_ = 0.71 e Å^−3^
Δρ_min_ = −0.34 e Å^−3^

### 

Data collection: *SMART* (Bruker, 1997 ▶); cell refinement: *SAINT* (Bruker, 1997 ▶); data reduction: *SAINT*; program(s) used to solve structure: *SHELXS97* (Sheldrick, 2008 ▶); program(s) used to refine structure: *SHELXL97* (Sheldrick, 2008 ▶); molecular graphics: *SHELXTL* (Sheldrick, 2008 ▶); software used to prepare material for publication: *SHELXTL*.

## Supplementary Material

Crystal structure: contains datablock(s) global, I. DOI: 10.1107/S1600536811028029/hb5903sup1.cif

Structure factors: contains datablock(s) I. DOI: 10.1107/S1600536811028029/hb5903Isup2.hkl

Supplementary material file. DOI: 10.1107/S1600536811028029/hb5903Isup3.cml

Additional supplementary materials:  crystallographic information; 3D view; checkCIF report

## Figures and Tables

**Table 1:** Hydrogen-bond geometry (Å, °)

*D*—H⋯*A*	*D*—H	H⋯*A*	*D*⋯*A*	*D*—H⋯*A*
N3—H3*A*⋯S2^i^	0.86	2.48	3.3195 (17)	166
N1—H1*A*⋯S2^ii^	0.86	2.72	3.430 (2)	141

Symmetry codes: (i) 
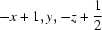
; (ii) 
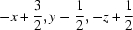
.

**Fig. 1.**
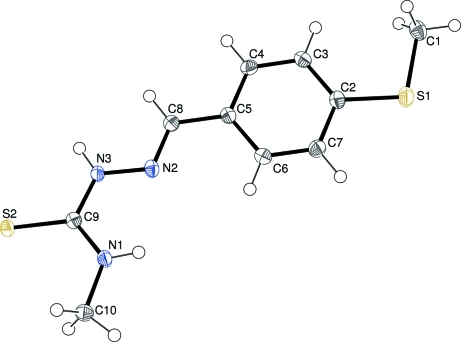
The structure of the title compound showing 50% probability displacement ellipsoids.

## supplementary crystallographic information

### Experimental

A mixture of 4-methylthiosemicarbazide (0.1 mol), and 4-(methylthio)benzaldehyde (0.1 mol) was stirred in refluxing ethanol (20 mL) for 4 h to afford the title compound (0.076 mol, yield 76%).  Colourless blocks of the title compound were obtained by recrystallization from ethanol at room temperature.

### Refinement

The absolute structure was indeterminate in the present study. H atoms were fixed geometrically and allowed to ride on their attached atoms, with C—H distances = 0.93–0.97 Å; N—H = 0.86Å and with *U*_iso_(H) = 1.2*U*_eq_(C,*N*) or 1.5*U*_eq_(C_methyl_).

### Crystal data

**Table:**

C_10_H_13_N_3_S_2_	*F*(000) = 1008
*M**_r_* = 239.35	*D*_x_ = 1.362 Mg m^−^^3^
Monoclinic, *C*2/*c*	Mo *K*α radiation, λ = 0.71073 Å
*a* = 14.123 (3) Å	Cell parameters from 2368 reflections
*b* = 7.7789 (16) Å	θ = 2.9–27.5°
*c* = 21.384 (4) Å	µ = 0.43 mm^−^^1^
β = 96.31 (3)°	*T* = 293 K
*V* = 2335.1 (8) Å^3^	Bar, colorless
*Z* = 8	0.23 × 0.18 × 0.17 mm

### Data collection

**Table:**

Bruker SMART CCD diffractometer	2368 reflections with *I* > 2σ(*I*)
Radiation source: fine-focus sealed tube	*R*_int_ = 0.043
graphite	θ_max_ = 27.5°, θ_min_ = 3.0°
phi and ω scans	*h* = −18→18
10756 measured reflections	*k* = −10→10
2680 independent reflections	*l* = −27→27

### Refinement

**Table:**

Refinement on *F*^2^	Primary atom site location: structure-invariant direct methods
Least-squares matrix: full	Secondary atom site location: difference Fourier map
*R*[*F*^2^ > 2σ(*F*^2^)] = 0.057	Hydrogen site location: inferred from neighbouring sites
*wR*(*F*^2^) = 0.170	H-atom parameters constrained
*S* = 1.35	*w* = 1/[σ^2^(*F*_o_^2^) + (0.1*P*)^2^] where *P* = (*F*_o_^2^ + 2*F*_c_^2^)/3
2680 reflections	(Δ/σ)_max_ = 0.001
136 parameters	Δρ_max_ = 0.71 e Å^−^^3^
0 restraints	Δρ_min_ = −0.34 e Å^−^^3^

### Special details

**Table:**

Geometry. All esds (except the esd in the dihedral angle between two l.s. planes) are estimated using the full covariance matrix. The cell esds are taken into account individually in the estimation of esds in distances, angles and torsion angles; correlations between esds in cell parameters are only used when they are defined by crystal symmetry. An approximate (isotropic) treatment of cell esds is used for estimating esds involving l.s. planes.
Refinement. Refinement of F^2^ against ALL reflections. The weighted R-factor wR and goodness of fit S are based on F^2^, conventional R-factors R are based on F, with F set to zero for negative F^2^. The threshold expression of F^2^ > 2sigma(F^2^) is used only for calculating R-factors(gt) etc. and is not relevant to the choice of reflections for refinement. R-factors based on F^2^ are statistically about twice as large as those based on F, and R- factors based on ALL data will be even larger.

### Fractional atomic coordinates and isotropic or equivalent isotropic displacement parameters (Å^2^)

**Table:**

	*x*	*y*	*z*	*U*_iso_*/*U*_eq_	
S2	0.62610 (3)	0.59132 (7)	0.31409 (2)	0.0160 (2)	
S1	0.65713 (4)	−0.10944 (9)	−0.11046 (3)	0.0285 (2)	
N3	0.59872 (11)	0.4593 (3)	0.19951 (7)	0.0165 (4)	
H3A	0.5447	0.5113	0.1957	0.020*	
N2	0.62552 (12)	0.3592 (2)	0.15110 (7)	0.0170 (4)	
N1	0.74248 (12)	0.3948 (3)	0.25443 (8)	0.0180 (4)	
H1A	0.7538	0.3359	0.2221	0.022*	
C9	0.65908 (13)	0.4741 (3)	0.25306 (9)	0.0152 (4)	
C2	0.62751 (14)	0.0364 (3)	−0.05305 (9)	0.0189 (4)	
C5	0.59070 (13)	0.2501 (3)	0.04735 (8)	0.0156 (4)	
C7	0.69010 (14)	0.0413 (3)	0.00246 (9)	0.0203 (5)	
H7A	0.7441	−0.0280	0.0063	0.024*	
C3	0.54653 (14)	0.1405 (3)	−0.05812 (9)	0.0191 (4)	
H3B	0.5048	0.1399	−0.0949	0.023*	
C6	0.67274 (13)	0.1475 (3)	0.05149 (9)	0.0181 (4)	
H6A	0.7158	0.1510	0.0876	0.022*	
C8	0.56888 (13)	0.3578 (3)	0.09981 (9)	0.0166 (4)	
H8A	0.5142	0.4253	0.0961	0.020*	
C10	0.81565 (14)	0.4023 (3)	0.30768 (9)	0.0197 (5)	
H10A	0.8697	0.3356	0.2986	0.030*	
H10B	0.7909	0.3565	0.3443	0.030*	
H10C	0.8347	0.5195	0.3153	0.030*	
C4	0.52844 (13)	0.2450 (3)	−0.00809 (9)	0.0185 (4)	
H4A	0.4739	0.3127	−0.0116	0.022*	
C1	0.56946 (16)	−0.0720 (3)	−0.17628 (10)	0.0233 (5)	
H1B	0.5809	−0.1476	−0.2101	0.035*	
H1C	0.5735	0.0452	−0.1899	0.035*	
H1D	0.5071	−0.0934	−0.1641	0.035*	

### Atomic displacement parameters (Å^2^)

**Table:**

	*U*^11^	*U*^22^	*U*^33^	*U*^12^	*U*^13^	*U*^23^
S2	0.0166 (3)	0.0151 (3)	0.0165 (3)	−0.00181 (16)	0.0024 (2)	−0.00314 (17)
S1	0.0262 (4)	0.0342 (4)	0.0247 (3)	0.0075 (2)	0.0007 (2)	−0.0115 (2)
N3	0.0154 (8)	0.0176 (10)	0.0163 (8)	0.0021 (6)	0.0008 (6)	−0.0041 (7)
N2	0.0205 (8)	0.0147 (10)	0.0163 (8)	−0.0016 (7)	0.0041 (6)	−0.0018 (7)
N1	0.0169 (8)	0.0198 (11)	0.0170 (8)	0.0007 (6)	0.0009 (6)	−0.0052 (6)
C9	0.0168 (9)	0.0116 (11)	0.0172 (9)	−0.0041 (7)	0.0026 (6)	0.0002 (7)
C2	0.0205 (10)	0.0183 (12)	0.0184 (9)	−0.0014 (8)	0.0046 (7)	−0.0013 (8)
C5	0.0206 (9)	0.0118 (11)	0.0147 (8)	−0.0031 (7)	0.0035 (6)	0.0016 (7)
C7	0.0170 (9)	0.0220 (13)	0.0220 (10)	0.0012 (8)	0.0031 (7)	−0.0003 (8)
C3	0.0208 (10)	0.0200 (13)	0.0161 (9)	−0.0015 (8)	−0.0003 (7)	0.0002 (8)
C6	0.0187 (10)	0.0185 (12)	0.0168 (9)	−0.0018 (8)	0.0008 (7)	0.0009 (8)
C8	0.0179 (9)	0.0145 (12)	0.0175 (9)	−0.0015 (7)	0.0021 (7)	0.0006 (8)
C10	0.0157 (10)	0.0222 (13)	0.0207 (10)	−0.0005 (7)	−0.0008 (7)	−0.0020 (8)
C4	0.0198 (10)	0.0165 (12)	0.0190 (9)	0.0011 (7)	0.0009 (7)	0.0026 (8)
C1	0.0310 (11)	0.0201 (13)	0.0194 (10)	−0.0045 (8)	0.0049 (8)	−0.0019 (8)

### Geometric parameters (Å, °)

**Table:**

S2—C9	1.699 (2)	C5—C8	1.460 (3)
S1—C2	1.756 (2)	C7—C6	1.378 (3)
S1—C1	1.793 (2)	C7—H7A	0.9300
N3—C9	1.355 (2)	C3—C4	1.389 (3)
N3—N2	1.381 (2)	C3—H3B	0.9300
N3—H3A	0.8600	C6—H6A	0.9300
N2—C8	1.285 (2)	C8—H8A	0.9300
N1—C9	1.327 (3)	C10—H10A	0.9600
N1—C10	1.452 (3)	C10—H10B	0.9600
N1—H1A	0.8600	C10—H10C	0.9600
C2—C3	1.396 (3)	C4—H4A	0.9300
C2—C7	1.401 (3)	C1—H1B	0.9600
C5—C4	1.397 (3)	C1—H1C	0.9600
C5—C6	1.402 (3)	C1—H1D	0.9600
			
C2—S1—C1	104.29 (10)	C2—C3—H3B	120.1
C9—N3—N2	118.85 (16)	C7—C6—C5	120.56 (17)
C9—N3—H3A	120.6	C7—C6—H6A	119.7
N2—N3—H3A	120.6	C5—C6—H6A	119.7
C8—N2—N3	116.64 (17)	N2—C8—C5	119.77 (18)
C9—N1—C10	123.52 (17)	N2—C8—H8A	120.1
C9—N1—H1A	118.2	C5—C8—H8A	120.1
C10—N1—H1A	118.2	N1—C10—H10A	109.5
N1—C9—N3	116.98 (18)	N1—C10—H10B	109.5
N1—C9—S2	123.37 (15)	H10A—C10—H10B	109.5
N3—C9—S2	119.65 (15)	N1—C10—H10C	109.5
C3—C2—C7	119.05 (19)	H10A—C10—H10C	109.5
C3—C2—S1	125.22 (15)	H10B—C10—H10C	109.5
C7—C2—S1	115.70 (16)	C3—C4—C5	121.24 (18)
C4—C5—C6	118.41 (17)	C3—C4—H4A	119.4
C4—C5—C8	120.17 (17)	C5—C4—H4A	119.4
C6—C5—C8	121.41 (17)	S1—C1—H1B	109.5
C6—C7—C2	120.85 (19)	S1—C1—H1C	109.5
C6—C7—H7A	119.6	H1B—C1—H1C	109.5
C2—C7—H7A	119.6	S1—C1—H1D	109.5
C4—C3—C2	119.87 (18)	H1B—C1—H1D	109.5
C4—C3—H3B	120.1	H1C—C1—H1D	109.5

### Hydrogen-bond geometry (Å, °)

**Table:**

*D*—H···*A*	*D*—H	H···*A*	*D*···*A*	*D*—H···*A*
N3—H3A···S2^i^	0.86	2.48	3.3195 (17)	166
N1—H1A···S2^ii^	0.86	2.72	3.430 (2)	141

Symmetry codes: (i) −*x*+1, *y*, −*z*+1/2; (ii) −*x*+3/2, *y*−1/2, −*z*+1/2.
